# Supplementary material for: Chytrid fungi distribution and co-occurrence with diatoms correlate with sea ice melt in the Arctic Ocean
Source: Commun Biol. 2020 Apr 21;3:183. doi: 10.1038/s42003-020-0891-7 (PMC7174370; doi:10.1038/s42003-020-0891-7)
Supplement: Supplementary file 1 — Supplementary Information [file 42003_2020_891_MOESM1_ESM.pdf]

Supplementary Figures

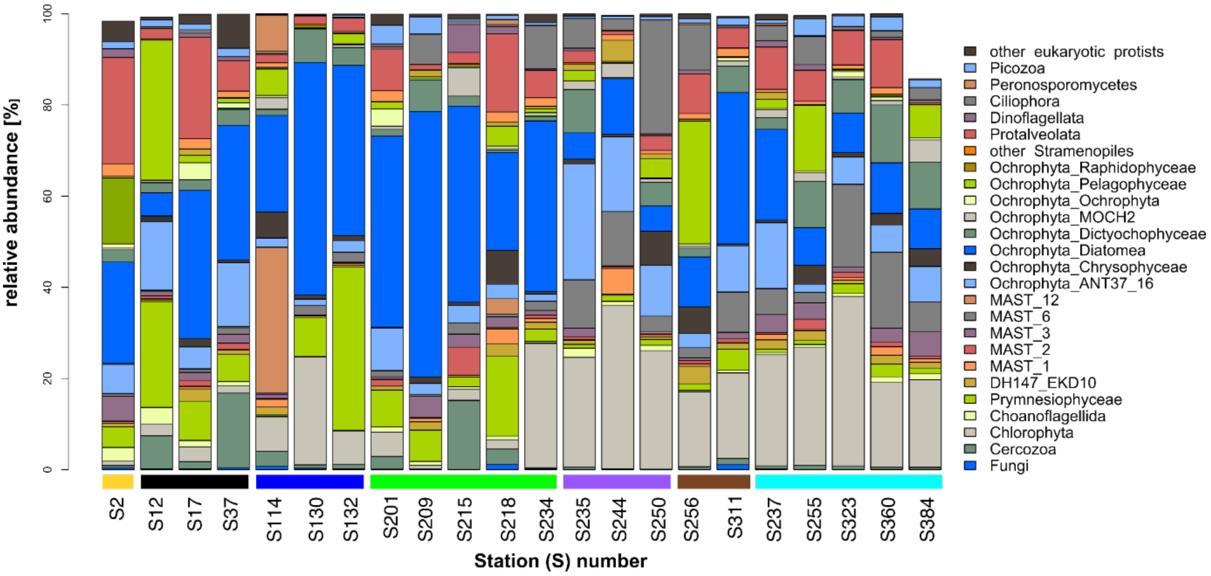

Supplementary Figure 1. Protist community composition across the sampling sites based on the V4 amplicon sequencing. The key indicates the taxonomic groups identified. The different oceanographic regions are illustrated as set of color bars across the X axis. The color codes are as follows: Norwegian Sea (orange), West Spitsbergen Current (black), East Greenland Current (blue), Barents Sea (green), Kara Sea (purple), Laptev Sea (brown) and under sea ice samples (light blue).

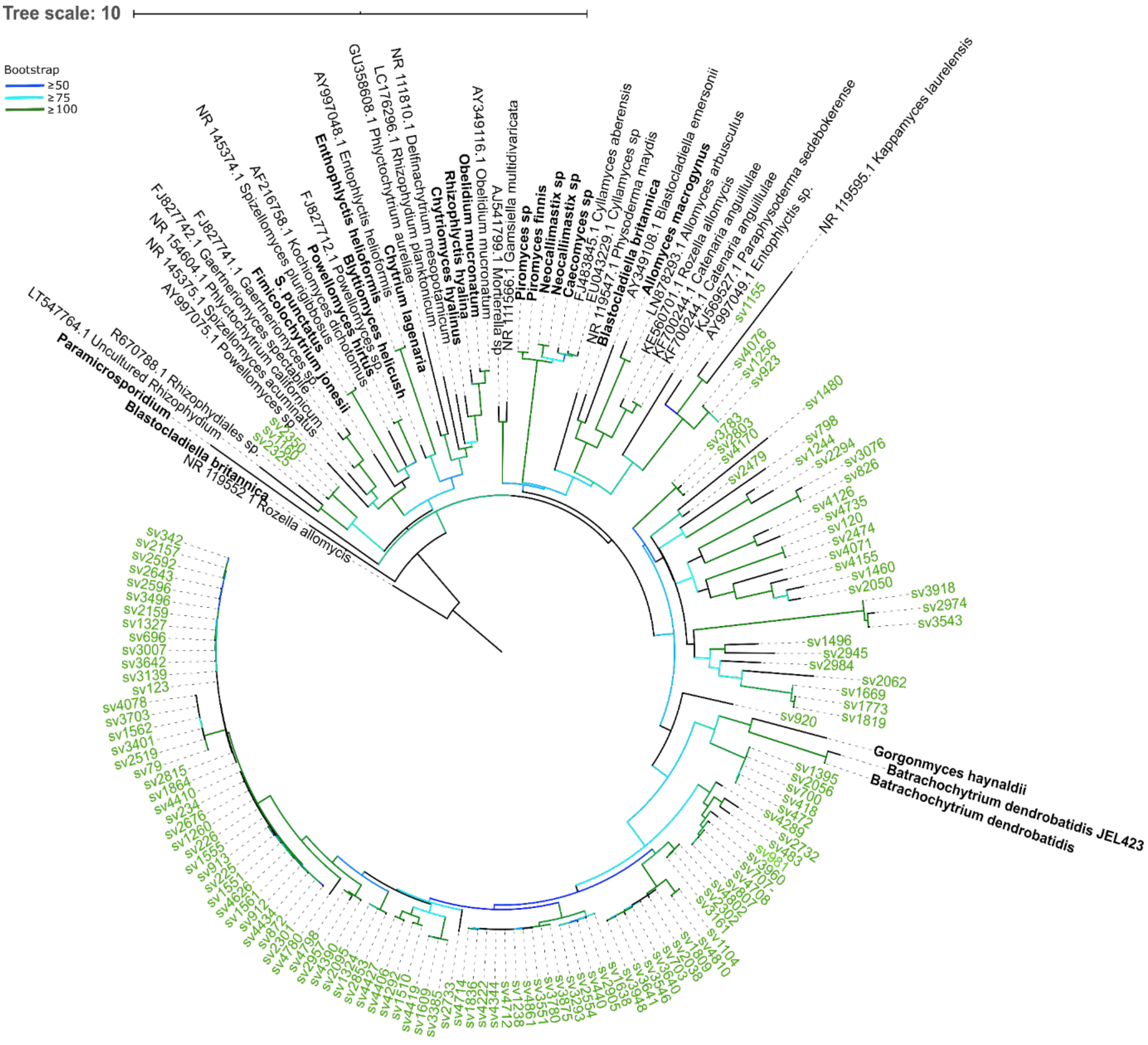

Supplementary Figure 2. Taxonomic classification of all chytrid sequence variants from the ITS2 data (green). The phylogenetic tree was computed based on the maximum likelihood method in IQ-Tree including 1000 bootstrap replicates r from a masked alignment of 177 taxa and 476 characters. Bold taxa refer to whole genome sequences recovered from public access databases i.e. Cryptomycota, Blastocladiomycota and Chytridiomycota accessed through the JGI genome portal and Ensembl Fungi.

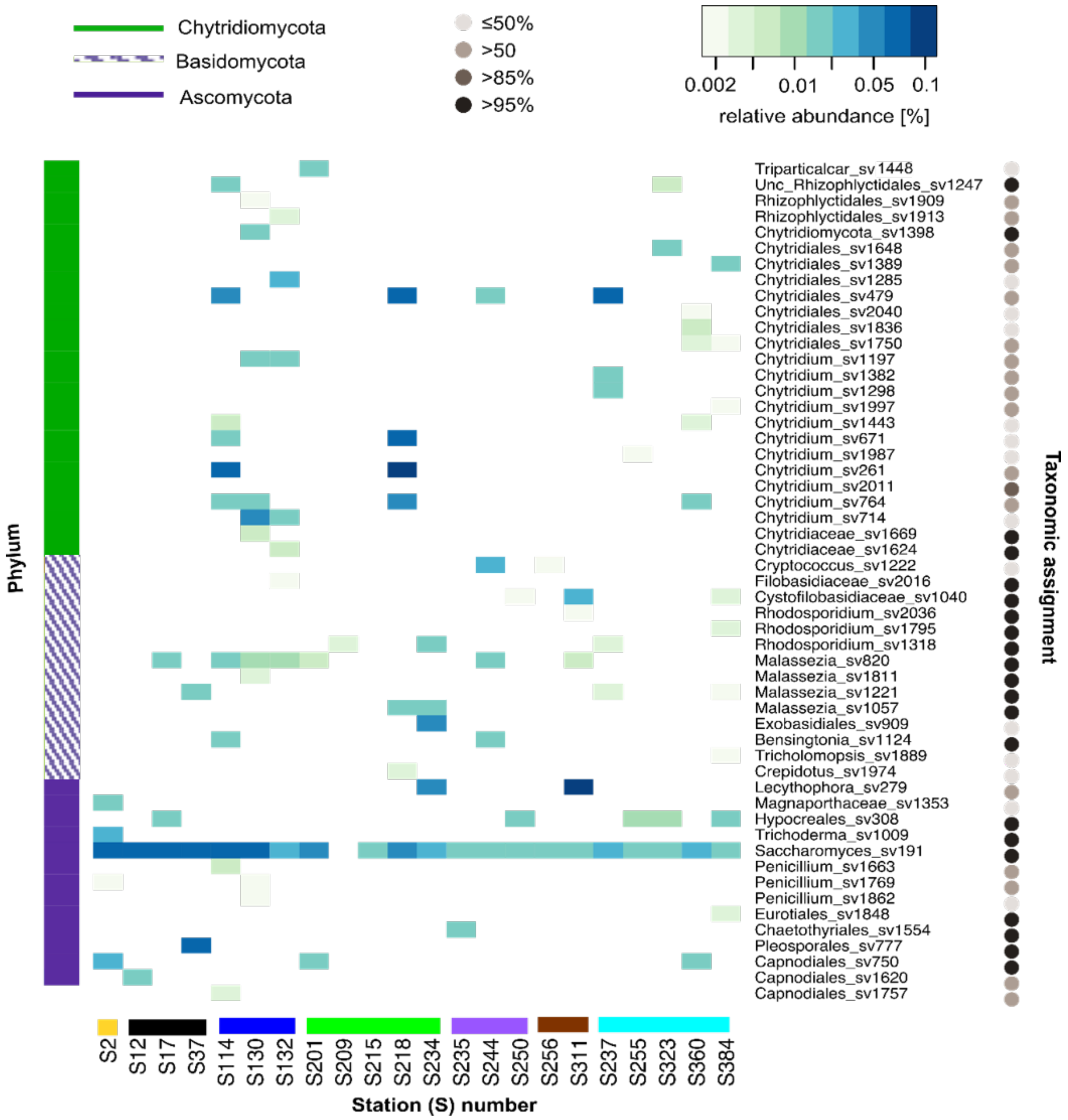

Supplementary Figure 3. Fungal community composition (Ascomycota, Basidiomycota and Chytridiomycota) across the sampling sites based on the V4 amplicon sequencing. The different oceanographic regions are shown as a colored bars across the x axis. The color codes are as follows: Norwegian Sea (orange), West Spitsbergen Current (black), East Greenland Current (blue), Barents Sea (green), Kara Sea (purple), Laptev Sea (brown) and under sea ice samples (light blue). Dots down the Y- axis indicate % sequence identify to the taxonomic assignment from DADA2 analysis.

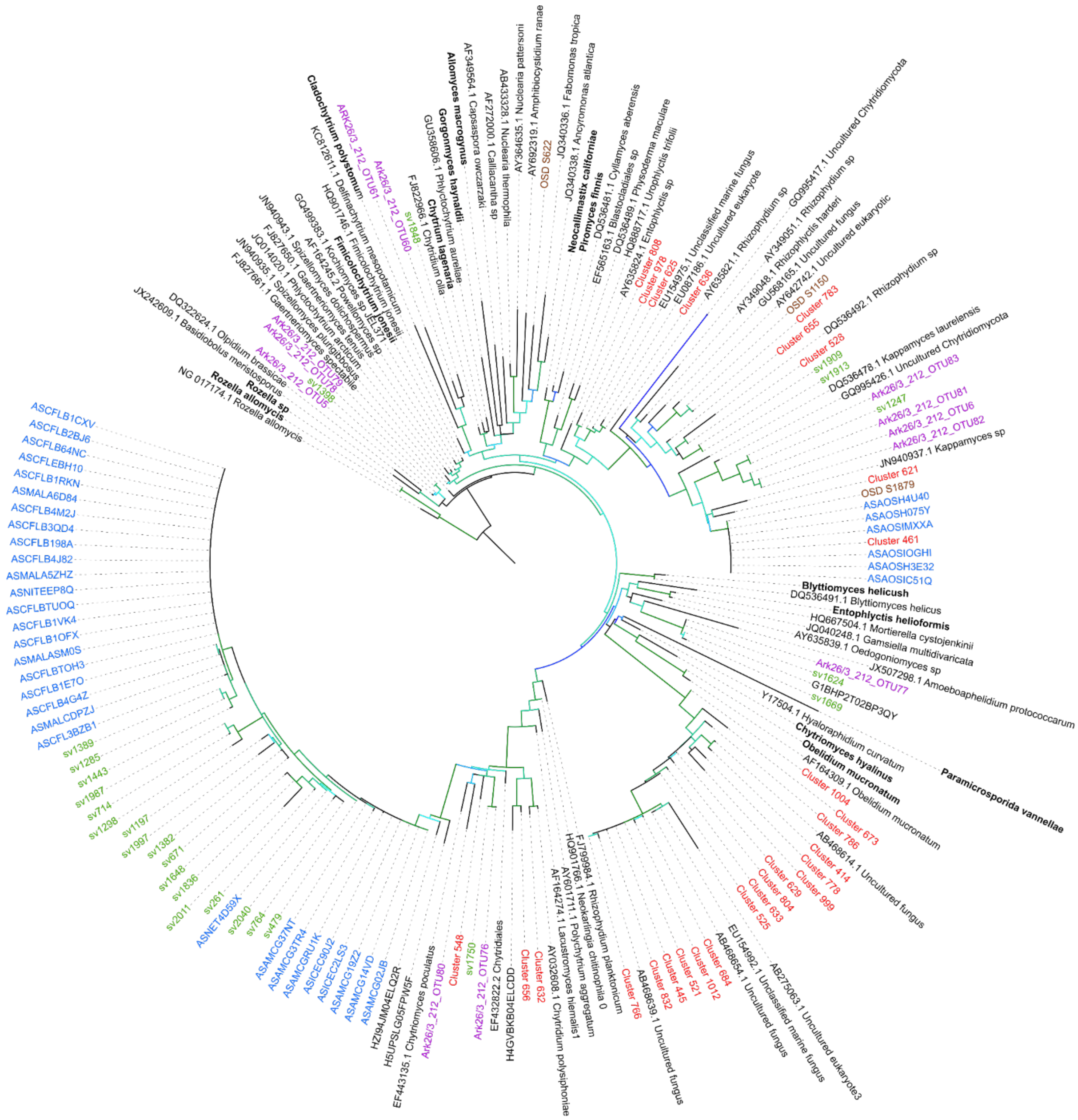

Supplementary Figure 4. Taxonomic placement of three Ocean Sampling Day (OSD) chytrid sequence variants (brown), the chytrid V4 sequence data generated as part of this study and V4 sequences recovered from fungal genome projects. The phylogenetic tree was computed based on the maximum likelihood method in IQ-Tree including 1000 bootstrap replicates from a masked alignment of 175 taxa and 383 characters. For the sample color code please see Figure 5. Bold taxa refer to whole genome sequences recovered from public access databases i.e. Cryptomycota, Blastocladiomycota and Chytridiomycota accessed through the JGI genome portal and Ensembl Fungi.
