# Supplementary material for: Chytrid fungi distribution and co-occurrence with diatoms correlate with sea ice melt in the Arctic Ocean
Source: Commun Biol. 2020 Apr 21;3:183. doi: 10.1038/s42003-020-0891-7 (PMC7174370; doi:10.1038/s42003-020-0891-7)
Supplement: Supplementary file 2 — Description of Additional Supplementary Files [file 42003_2020_891_MOESM2_ESM.pdf]

Description of additional supplementary files

Supplementary data 1.

The Supplementary Data 1 excel file contains the results of all DNA-seq analyses used to generate figures in which data values are shown.

- 1. Sheet: Relative abundance data used to generate Figure1b.
- 2. Sheet: Data points used to generate the NMDS plots in Figure 2 (including meta data).
- 3. Sheet: Relative abundance data of diatoms used to generate Figure 3.
- 4. Sheet: Data points for fungal group distribution Figure 4.
- 5. Sheet: Data points used to generate co-occurrence plot in Figure 6a.
- 7. Sheet: Data points for the correlation analysis on the basis of the ‘Ocean Sampling Day’ dataset used to generate Figure 8
- 8. Sheet: P-values for the correlation analysis on the basis of the ‘Ocean Sampling Day’ dataset used to generate Figure 8.
