# Supplementary material for: Chytrid fungi distribution and co-occurrence with diatoms correlate with sea ice melt in the Arctic Ocean
Source: Commun Biol. 2020 Apr 21;3:183. doi: 10.1038/s42003-020-0891-7 (PMC7174370; doi:10.1038/s42003-020-0891-7)
Supplement: Supplementary file 4 — Reporting Summary [file 42003_2020_891_MOESM4_ESM.pdf]

natureresearch

Corresponding author(s): Estelle S. Kilias

Last updated by author(s): Jan 8, 2020

Reporting Summary

Nature Research wishes to improve the reproducibility of the work that we publish. This form provides structure for consistency and transparency in reporting. For further information on Nature Research policies, see Authors & Referees and the Editorial Policy Checklist.

Statistics

For all statistical analyses, confirm that the following items are present in the figure legend, table legend, main text, or Methods section.

n/a	Confirmed	
☐	☒	The exact sample size (n) for each experimental group/condition, given as a discrete number and unit of measurement
☐	☒	A statement on whether measurements were taken from distinct samples or whether the same sample was measured repeatedly
☒	☐	The statistical test(s) used AND whether they are one- or two-sided<br>*Only common tests should be described solely by name; describe more complex techniques in the Methods section.*
☒	☐	A description of all covariates tested
☒	☐	A description of any assumptions or corrections, such as tests of normality and adjustment for multiple comparisons
☒	☐	A full description of the statistical parameters including central tendency (e.g. means) or other basic estimates (e.g. regression coefficient) AND variation (e.g. standard deviation) or associated estimates of uncertainty (e.g. confidence intervals)
☒	☐	For null hypothesis testing, the test statistic (e.g. F, t, r) with confidence intervals, effect sizes, degrees of freedom and P value noted<br>*Give P values as exact values whenever suitable.*
☒	☐	For Bayesian analysis, information on the choice of priors and Markov chain Monte Carlo settings
☒	☐	For hierarchical and complex designs, identification of the appropriate level for tests and full reporting of outcomes
☒	☐	Estimates of effect sizes (e.g. Cohen's d, Pearson's r), indicating how they were calculated

Our web collection on statistics for biologists contains articles on many of the points above.

Software and code

Policy information about availability of computer code

Data collection	The data (raw sequence reads) were collected/processed using commercial R package Dada2 (version 1.12.1) using standart filtering parameters (e.g. maxN=0 or truncQ=2) as stated on the website.
Data analysis	Throughout the manuscript, data analysis is based on widely used software that has been described previously. The following software has been used in chronological order: R (version 3.2.3); R package vegan (2.5-5), ade4 (1.7-13); heatmap3 (1.1.6), robcompositions (2.1.0); corrplot (0.84); SparCC; IQ tree (1.6.6.)

For manuscripts utilizing custom algorithms or software that are central to the research but not yet described in published literature, software must be made available to editors/reviewers. We strongly encourage code deposition in a community repository (e.g. GitHub). See the Nature Research guidelines for submitting code & software for further information.

Data

Policy information about availability of data

All manuscripts must include a data availability statement. This statement should provide the following information, where applicable:

- Accession codes, unique identifiers, or web links for publicly available datasets
- A list of figures that have associated raw data
- A description of any restrictions on data availability

All sequences have been deposited in the NCBI Sequence Read Arhive (SRA) under the accession number PRJNA561496.

Field-specific reporting

Please select the one below that is the best fit for your research. If you are not sure, read the appropriate sections before making your selection.

☐ Life sciences ☐ Behavioural & social sciences ☒ Ecological, evolutionary & environmental sciences

For a reference copy of the document with all sections, see nature.com/documents/nr-reporting-summary-flat.pdf

Ecological, evolutionary & environmental sciences study design

All studies must disclose on these points even when the disclosure is negative.

Study description	The study investigates the distribution and diversity of marine-derived fungi in the northern hemisphere (Norwegian Sea, Fram Strait and Arctic Ocean) in relation to environmental factors and potential host candidates such as diatoms.
Research sample	A total of 22 environmental water samples (deep chlorophyll max and under ice surface water) have been taken along the course of two cruise legs in 2012 (ARK XXVII-2/-3) of the RV Polarstern
Sampling strategy	In detail, all water samples have been taken with the CTD rosette, sampling the deep chlorophyll max as well as the under ice surface water layer. Two liters were filtered onto a 0.4 micrometer filter not exceeding 200 mbar to reduce the danger of cell breakage and stored at -80 C.
Data collection	Meta data such as temperature and salinity were recorded by the profilers attached to the CTD, while sea ice concentration/coverage was based on visual estimates. Amplicon sequence data were retrieved by paired-end sequencing on the MiSeq Illumina platform after DNA was extracted using the DNA Powersoil DNA extraction kit from MOBIO. For library preparation the Nextera XT DNA library kit was used. The concentration and quality of the library was quantified before sequencing by Qubit and qPCR.
Timing and spatial scale	Samples have been taken between 2012/06/17 and 2012/09/28. In this regard, the sampling area covers samples in the Norwegian Sea, Fram Strait, Barents Sea, Kara Sea and Laptev Sea.
Data exclusions	No data were excluded from the analysis
Reproducibility	The sequence data represent snapshots of the eukaryotic microbial community on the day of sampling. Fast changing environmental conditions in the Arctic Ocean don't support for the reproducibility of the data in future biological samples. However, reproducibility in form of re-sequencing of the extracted DNA from 2012 is possible with potential small variations in the representation of e.g. the rare biosphere.
Randomization	Samples were grouped based on sampling depth (DCM vs. under ice surface water) and geographic location. Sequence data were grouped into sequence variants using the R package Dada2 and the implemented algorithm. Taxonomic assignment was carried out based on the SILVA database (version 128) and the implemented naive Bayesian classifier (97 % identity).
Blinding	Blinding was not relevant for this study.

Did the study involve field work? ☒ Yes ☐ No

Field work, collection and transport

Field conditions	The expedition was conducted in 2012 and covered a wide geographic range. Many sampling stations were influenced by sea ice melt conditions. In general, the water temperature ranged between -1.8 C (Arctic Ocean) and +8.97 C (Norwegian Sea). As such the salinity showed a wide spectrum including salinities between 30.54 and 35.09 PSU. Out of 22 sampling stations 7 showed no sea ice, while 15 showed different sea ice concentrations.
Location	Station (S): S2 (lat: 67.65; lon:6.85; 15 meter); S12 (lat: 79.383333; lon: 9.816667; 30 meter); S17 (lat: 79.383333; lon: 8.816667; 32 meter); S37 (lat: 79.383333; lon: 5.15; 10 meter); S114 (lat: 79.25; lon: -13.3; 30 meter); S130 (lat: 79.383333; lon: -4.983333; 20 meter); S132 (lat: 79.36667; lon: -3.15; 12 meter); S201 (lat: 81; lon: 30.63333; 30 meter); S209 (lat: 81.816667; lon: 30.28333; 32 meter); S215 (lat: 82.816667; lon: 30; 17 meter); S218 (lat: 83.65; lon: 30.08333; 50 meter); S234 (lat: 84.65 lon: 939.78333; 22 meter); S235 (lat: 84.53333; lon: 60.1; 26 meter); S244 (lat: 83.316667; lon: 75.15; 30 meter); S250 (lat: 83.966667; lon: 87.75; 30 meter); S256 (lat: 83.116667; lon: 109.983333; 20 meter); S311 (lat: 77.65; lon: 118.316667; 20 meter); S237 (lat: 84.6; lon: 77.1; under ice surface); S255 (lat: 83.266667; lon: 109.1; under ice surface); S323 (lat: 82.55; lon: 131.216667; under ice surface); S360 (lat: 89.383333; lon: 58.13333; under ice surface); S384 (lat: 84.63333; lon: 17.75; under ice surface).
Access and import/export	Access to the sampling sites was provided by the cooperation with the Alfred Wegener Institute and the attendance of the expedition on the ice breaker RV Polarstern. Filtered frozen water samples were stored until the end of the expedition at -80 C and later sent on dry ice to the University of Exeter where they have been stored at -80 C until processing.
Disturbance	No disturbance was caused by the expedition

Reporting for specific materials, systems and methods

We require information from authors about some types of materials, experimental systems and methods used in many studies. Here, indicate whether each material, system or method listed is relevant to your study. If you are not sure if a list item applies to your research, read the appropriate section before selecting a response.

Materials & experimental systems

n/a	Involved in the study
☒	☐ Antibodies
☒	☐ Eukaryotic cell lines
☒	☐ Palaeontology
☒	☐ Animals and other organisms
☒	☐ Human research participants
☒	☐ Clinical data

Methods

n/a	Involved in the study
☒	☐ ChIP-seq
☒	☐ Flow cytometry
☒	☐ MRI-based neuroimaging
